# Introducing a Mobile Learning Model in Medical Education during COVID-19; A Critical Review

**DOI:** 10.30476/JAMP.2022.93494.1534

**Published:** 2022-07-01

**Authors:** MASOMEH KALANTARION, MOHAMMAD-MEHDI SADOUGHI, SOLEIMAN AHMADY, PER KALLESTRUP, MARZIEH KATIBEH, NASRIN KHAJEALI

**Affiliations:** 1 Department of Medical Education, Virtual School of Medical Education and Management, Shahid Beheshti University of Medical Sciences, Tehran, Iran; 2 Ophthalmic Research Center, Research Institute for Ophthalmology and Vision Science, Shahid Beheshti University of Medical Sciences, Tehran, Iran; 3 Centre for Global Health, Department of Public Health, Aarhus University, Aarhus, Denmark; 4 Department of Medical Education, Ahvaz Jundishapur University of Medical Sciences, Ahvaz, Iran

**Keywords:** Computers, Handheld, Learning, SARS-CoV-2

## Abstract

**Introduction:**

Mobile learning is one of the innovative teaching techniques that help medical students gain knowledge and skills. One of the factors that expanded the use of this strategy was the COVID-19 pandemic. However, the educational pedagogy of such technology has been neglected. This article aimed to critically review available mobile learning models in medical education to suggest a comprehensive model in the field of mobile learning.

**Methods:**

We conducted this critical review based on the five steps of the Carnwell and Daly method. For a comprehensive systematic search from 2000 to April 2021, the following keywords were used: Personal Digital Assistant, m learning, Mobile learning, Ubiquitous learning, U learning, medical students, and medical education. 3176 studies in PubMed, Scopus, ERIC, Magiran, and Web of Science were identified. In total, 8 articles entered the study.

**Results:**

Eight models of mobile learning in medical education were identified. The key features of each model were extracted and integrated into the new model for the successful design and implementation of mobile learning. This model includes three main elements of mobile learning: 1-stakeholders, 2-interaction, and 3-technology, which are influenced by external factors including Mobiquette, legitimacy, and awareness.

**Conclusion:**

The results of this study are an important contribution to the knowledge collection in mobile learning in medical education. We introduced a comprehensive model of mobile learning including specific characteristics of strategies in the context of medical education.

## Introduction

Recently, higher education has undergone extensive changes due to technological advances ( 1 ). Following the increased use of modern communication technologies, traditional teaching methods using these technologies have introduced the concept of e-Learning ( 2 ). The emergence of mobile technologies such as notebooks, tablets, and smartphones in e-learning, has led to the term and concept of mobile learning (m-Learning) ( 3 ). The main characteristic of m‑Learning is Anytime Anywhere Learning ( 4 ).

There are many definitions of m-Learning. In 2003, Brown defined m-Learning as an extension of e-learning ( 2 ). Sharples stated that m-Learning was a way to support learning out of the classroom in the interactions between students ( 5 ). Harden, a world leader in medical education, expanded m-Learning to include any kind of learning using mobile technologies created along with mobility by using flexible learning opportunities ( 6 ). The concept of m-learning was introduced by Alan Kay in the 1970s. He formed a group to develop a portable personal computer, the “Dynabook”. The project was unsuccessful because of the lack of technological support at the time ( 7 ). Mobile education has been practiced in organizations, institutions, and schools since 2000 ( 8 ). Today, research is performed worldwide on m-Learning ( 5 , 9 ).

The learners use mobile for their daily activities, so they are tend to apply them in their educations ( 10 ). This availability of mobile can enhance learning and promote the teacher’s role in solving the problems of each student ( 11 ). Another feature of mobile devices compared to traditional learning materials is their portability, which makes the students carry them easily and have more access to educational content ( 10 - 11 ).

Some evidence has indicated that m-Learning has the potential to improve the use of evidence-based decision-making ( 12 ). In the development of new teaching methods and learning resources, significant progress has been made in the availability of electronic resources and mobile devices. Recent reports indicate an increase in the use of mobile devices by the younger age groups with easy access to the Internet and applications relevant for medicine ( 13 ). Today, mobile devices are widely used by physicians during care to access up-to-date medical resources ( 14 ). Their use in both clinical practice and medical education is in line with the requirements of the General Medical Council (GMC) ( 12 ) and is generally considered to have many benefits for both teachers and students ( 15 ). 

Several prominent medical universities such as Oxford ( 16 ), Harvard ( 17 ), Johns Hopkins ( 18 ), Sydney ( 19 ), Tokyo ( 20 ), University of Leeds ( 21 ), Brighton and Sussex Medical School ( 22 ), and University of Melbourne ( 23 ) use a wide range of m-Learning strategies to optimize learning.

The COVID-19 pandemic caused an expansion in the use of m-Learning ( 24 , 25 ). During the pandemic, medical educational institutions have been forced to develop new ways to overcome challenges to traditional teaching ( 26 ). This critical pandemic reportedly deteriorated the quality of education. The educational system was forced to strengthen the use of creative teaching techniques ( 27 ). M-Learning is one of the innovative teaching techniques that helped medical students to gain technological skills, social skills, receive fast and timely feedback, and develop cooperative learning ( 28 ).

Several studies have been conducted to evaluate the practical application of mobile devices and computers in medical education; however, literature is scarce on the educational pedagogy needed to use this technology ( 29 , 30 ). If we have limited resources to deliver m-Learning, it needs to be theoretically justifiable ( 31 ). Today, research in the field of m-Learning has focused on the effectiveness and comparison of new and old technologies. 

There are several outstanding m-Learning models in higher education ( 32 ), but in medical education, models are limited. There is lack of research on when and how to use m-Learning effectively ( 33 ). Therefore, this article aimed to critically review m-Learning models in medical education and identify key elements for a comprehensive model.

## Methods

This study applied the method of critical review based on the ‘Five-phase method’ adopted by Carnwell and Daly . The five steps are: a) determining the scope of review, b) recognizing relevant information sources, c) reviewing the evidence, d) applying a general and critical perspective in writing, and e) concluding the literature for further studies ( 34 ). The researcher investigates the existing evidence in the field of a subject with a critical view and identifies knowledge gaps and proposes new studies in the field ( 35 ). Therefore, we identified m-Learning models in medical education to examine their important elements to achieve a comprehensive model.

According to Hart ( 36 ), a critical review should lead to the following conclusions:

What research has been done on m-Learning models in medical education and what are the gaps?

Which key elements should be considered for m-Learning model in the medical education system?

This review included reports and peer-reviewed articles related to m-Learning from 2000 to April 2021 that were retrieved in the databases PubMed, Scopus, ERIC, Magiran, and Web of Science.

The ontological search was keywords related to m-Learning: Personal Digital Assistant, m learning, Mobile learning, Ubiquitous learning, U learning, medical students, and medical education.

Titles and abstracts were screened by two independent researchers to determine the relevance. The full-text versions of the included materials were reviewed. In case of doubts regarding eligibility, a third researcher was consulted to resolve any disagreements (Table 1).

**Table 1:** Summary of inclusion and exclusion criteria

Study characteristics	Inclusion criteria	Exclusion criteria
Population	Medical students	Other health professionals
Subject	Texts related to key elements of m- learning in medical education	-
Language	English and Persian	-
Time	2000-2021	-
Type of studies	No limitation	-

Gray literature identifies by hand-searching through conference proceedings, theses, and abstracts.

**Figure 1:**
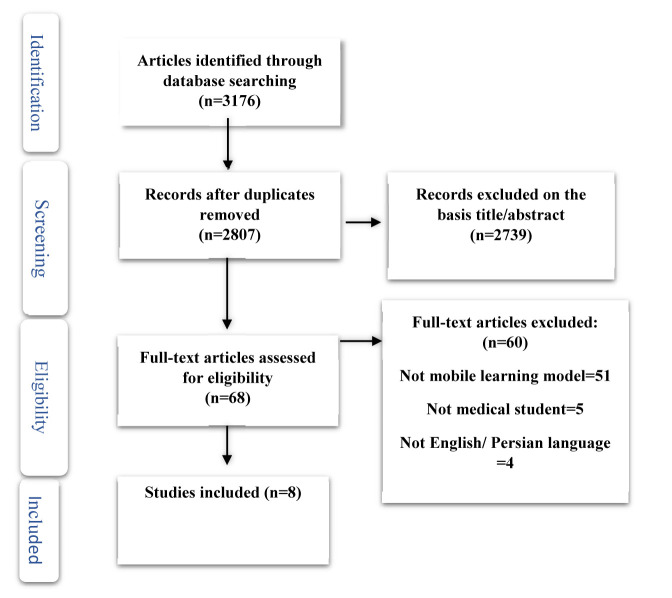
PRISMA diagram of the study selection results

The search identified 3176 articles. 2739 articles were removed since the title, keywords, or abstract did not demonstrate the desired concepts. At the eligibility step, 68 full texts of documents remained, and then 60 articles were excluded based on the inclusion/exclusion criteria. Finally, 8 articles were included (Figure 1).

Eight models of m-Learning in medical education were identified. The extracted data included authors, year of publication, country, model components, participants, sample, and outcomes (Table 2).

**Table 2:** Description of eight m-Learning models in medical education

Author's name	Publication year	Country	Type of study design	Model components	Participants	Sample	Outcome
Davies et al.	2012	United Kingdom	Mixed method	-External or internal elements	Medical students	387	Developed a model for m- learning in the clinical setting
				-Identify educational need			
				-Contextual learning			
				-Repetition			
				-Consolidation			
				-Positive and negative factors			
Briz-Ponce L et al.	2015	Spain	Quantitative study	-Perceived usefulness	Students and professionals	124	Design, implement and verify that the Technology Acceptance Model (TAM) can be employed within medical education
				-Perceived ease of use			
				-Attitude towards using technology			
				-Social Influence			
				-Facilitating conditions			
				-Self-efficacy			
				-Anxiety			
				-Behavioral intention to use the new technology			
				-Reliability			
				-Recommendation			
Joynes V et al.	2016	United Kingdom	Qualitative	-Maturity of learning	Medical students + clinical teachers	32 + 4	The developed conceptual framework for how the use of mobile resources can shape learning behaviors
				-Learning differently			
				-Learning legitimately			
				-Personalization			
				-Developing a professional identity			
Kohestani HR et al.	2018	Iran	Qualitative	-Motivational factors (negative and positive)	Medical students (from all five years) + Faculty member of university	23 + 5	The developed the model of m-Learning in medical education
				-Attitude			
				-Situational Reaction			
				-Usefulness perceived			
				-Reflection			
				-Behavioral intention			
Aliaño AM et al.	2019	Spain	Quantitative study	Gender	Health sciences student	370	The developed new model of technological acceptance based on the unified theory of acceptance and use of technology (UTAUT)
				Age			
				Performance Expectancy (PE) Effort Expectancy (EE) Social Influence (SI) Voluntariness to Use (VU) Facilitating Conditions (FC) Self-management of Learning (SL) Perceived Gratification (PG)			
				Behavioral Intention (BI)			
Lall P et al.	2019	United Kingdom	Qualitative study	-Device aspect	Medical sciences	47 Studies	Adapted FRAME model for medical and nursing education
				-Learner aspect – Social aspect			
				-Device usability			
				-Social technology			
				-Interaction learning			
				- Implementation			
Kucuk S et al.	2020	Turkey	Quantitative study	-Perceived usefulness	Medical sciences	376	The developed model explains medical students’ behavioral intention to use m-Learning
				-Perceived ease of use			
				-Instructor readiness			
				-Student readiness			
				-Attitude towards using technology			
				-Self-efficacy			
				-Learning autonomy			
				-Attitude			
				-Subjective norm			
				-Perceived behavioral control			
				-Behavioral intention			
Mosalanejad L et al.	2020	Iran	Mixed method	-Perceived usefulness	Medical students	150	The developed a new technology acceptance model
				-Need fulfillment students			
				-Attitudes			
				-Social factors			
				-Interactive factors			
				-Learning Factor			
				-Limitation Access to online resources			
				-Increasing virtual errors			
				-Cultural limitations			

### Ethics committee approval

This study was approved by the Research Ethics Committees of School of Medical Education affiliated to Shahid Behshti University of Medical Sciences, Tehran, Iran with the code of IR.SBMU.SME.REC.1400.026.

## Results

The final review included eight models, primarily rooted in developed countries. 

Davies et al. ( 33 ) developed m-Learning framework in a clinical setting including external or internal elements leading to the identification of an educational needs which could be met by using a mobile. Learning in a context with timely access helps the student to consolidate knowledge through repetition. Positive and negative factors such as negative social feedback, readiness, and acceptance of using IT may play a role at any stage of the learning process.

Briz-Ponce et al. ( 37 ) considered a framework to demonstrate that individual characteristics and external variables may significantly influence the use of m-Learning in medical education. Recommendation of mobile technology, self-efficacy in the use of technology, and positive attitude about the new technology are the main individual characteristics in m-Learning. External variables such as facilitating conditions (available resources), anxiety (lack of information), and social influences (impact on others) perceived usefulness and ease of use, and necessity of quality certification for apps indirectly affected the intention to use m-Learning.

Joynes et al. ( 21 ) also developed a conceptual model on how the use of mobile resources can shape learning behaviors in medical education. Five components emerged: ‘maturity of learning’ personalization, learning legitimately, developing professional identity, and learning differently.

"Maturity of learning" is related to how senior students demonstrated greater maturity in using resources than junior students. Another component, "personalization" is about students adapting the available resources to tailor their own needs. The concept of ‘learning legitimately’ is key to success of m-Learning. Participants indicated that legitimacy, as the mandatory nature of the program, has been a factor in encouraging them to use m-Learning.

"Developing professional identity", based on the participant's experience, is the use of mobile resources at the undergraduate level leaving a lasting impact after graduation and causing maturity in their behavioral patterns of learning in their work-life as health professionals.

The component "learning differently" was at the core of the model. Personalization, learning legitimately, maturity of learning, and developing professional identity revolved around the core component. M-Learning permits students and faculty to gain various learning experiences. One of these experiences improved their ability to "personalize" mobile resources for learning. Another experience was that the participants learned how to maturely use the mobile resource in the workplace over time.

Koohestani et al. ( 38 ) proposed a m-Learning model for medical students designed according to local conditions and contexts. This model consisted of five components such as motivational factors, attitude, situational reaction, perceived usefulness, reflection, and behavioral Intention. These five components are in an iterative process.

Motivational factors (negative and positive) cause negative and positive attitudes in students, which affect the behavioral intention and situational reaction. Students understand the benefits of using m-Learning in the learning path, which causes them to reflect, and ultimately this reflection affects motivational factors.

Aliano ( 39 ) designed a model for determining the factors affecting the acceptance and intention to use smartphones and tablets as learning resources in medical universities, as well as examining the relationships between these factors. In this model, age and gender were considered as the moderating variables. Perceived usefulness, perceived ease of use, facilitating conditions, and perceived gratification were independent variables and the behavioral intention was a dependent variable.

Perceived gratification means that working in an environment with mobile technologies results in improved motivation causing greater personal satisfaction. Thus, the process of learning becomes more enjoyable, provoking greater interest in the students. Some of these components are identical to the components of the model by Briz-Ponce ( 37 ), except for perceived gratification and socio-demographic data (age, gender).

One of the salient features of this model is that it examines the relationship between socio-demographic data and other components. Age and perceived usefulness had an inverse relation, similar to the perceived ease of use and the perceived gratification variable. 

The modified model FRAME for medical and nursing education context was introduced by Lall et al. ( 40 ). The FRAME model has three components: device, learner, and social examination of how aspects of mobile technology, together with learner capacities and social interaction, influence learning processes in an educational environment. These have something in common, such as device usability describing how learners related to the device. The second common point, social technology, is the intersection between the device and social aspects. The third model, the intersection between learner and social aspects, is named interaction learning.

In the modified model FRAME, social technology was changed to make up for the impact of mobile technologies on social interaction (with patients and the management of professional identity). They also added a circle that covers three circles named implementation. Insufficient institutional resources, lack of training and support, and limited planning and management of m-Learning seem to be the key to understanding how m-Learning for medical students might be implemented.

Kucuk ( 41 ) proposed a model based on the theory of planned behavior in a medical education context. Based on the theory of planned behavior (TPB), an individual’s attitude, subjective norms, and perceived behavioral control are determinants of behavioral intention. This model describes how medical students’ beliefs affect their intention to use m-Learning in their education.

In this model, perceived usefulness, which means personal beliefs that individuals obtain success in their performance when they use pertinent technology, affects the attitude to use m-Learning. Also, subjective norms, beliefs about whether most people agree or disagree with the behavior, are influenced by students’ readiness. Perceived behavioral control is mainly influenced by perceived self-efficacy. This means that if students make sure they use mobile apps, they will have behavioral control and intend to use it for m-Learning purposes. 

Mosalanejad ( 42 ) suggested a new technology acceptance model based on the TAM / FRAME models. In this model, perceived usefulness and needs fulfillment affect the students' attitudes towards using m-Learning. Attention to promoting social, interactive, and participatory factors can also influence the learners' decisions to use m-Learning. Access to online resources, increase in virtual errors, and cultural limitations are some of the barriers that affect the attitude and ultimately the intention to use mobile devices in education.

## Discussion

Eight m-Learning models were extracted from 8 reviewed articles and the key elements of each model were described. In this section, based on the fourth step in the method of Carnwell and Daly, the critics' views (if any) were reviewed, and at the end, our views are explained ( 34 ).

Davies and colleagues answered questions such as how medical students use mobile technologies. One of the strengths of this study is that m-Learning is used in formal medical education (in a clinical setting in the UK) and data were collected in both quantitative and qualitative ways ( 33 ). Another strength of this study is that the same device and resources were made available to all students. Lumsden et al. and Wallace et al. also endorsed this model as suitable for m-Learning in clinical education and believed that this model showed how the use of mobile devices has a good effect on learning ( 43 , 44 ). However, in the mentioned model, the participation of all stakeholders in education was not considered and only students were mentioned. The ease of the use of the mobile device, the use of m-Learning alongside traditional education, the features of mobile content, as well as educational design and student assessment were not mentioned. Ethical concerns about patients’ privacy and data security were not addressed.

The second model introduced by Briz-Ponce and colleagues attempts to provide insight into the factors that may affect the acceptance of mobile devices and applications by students and medical professionals in medical education ( 37 ). Few studies have specifically examined m-Learning in the field of medical education. This can be considered the first model that shows that personality traits, and external variables may have a significant impact on stakeholders to predict their behavioral intent.

Niazazari and colleagues acknowledged that in addition to the above points, the location and type of mobile device affected the acceptance of m-Learning ( 45 ). AL-Emran et al. also believed that many other factors still needed to be examined to confirm their effectiveness as external variables in this model ( 46 ). However, in designing this model, issues such as stakeholders, learning context, use of m- learning along with traditional education, educational design, features of mobile content, and also student assessment were not mentioned. In terms of technology, easy access to learning content, connection to the network, technical support, and the cost of equipment and facilities have not been considered.

In 2016, Joynes and colleagues developed a model for m-Learning by examining the views of students and educators on the impact of MBChB Mobile ( 21 ). In this model, mobile resources shape learning behaviors in the society and cause the individual growth of medical students. Koohestani and colleagues have also suggested that m-Learning may lead to valuable educational benefits ( 47 ). A salient feature of this model is that it is based on the experiences of a medical school and has been tested. Longitudinal data collection was also performed using a hybrid (quantitative-qualitative) approach. 

Another strength of this model is that it is not considered as an alternative to conventional methods. Instead, mobile resources align and complement the curriculum by adding different learning options. Thus, m-Learning integrates into the curriculum and is also a kind of blended learning creation. Endorsing this model, Green and colleagues believe that blended learning creates a rich and engaging experience for students ( 48 ).

In the mentioned model, only students and teachers were considered stakeholders. Although this model is based on practical faculty experiences, the features of content, educational design, and comprehensive evaluation methods used are not mentioned in the model. Also, in the implementation of this model, factors such as network connection and ease of access to content, technical support team, and costs have been considered, but in the m-Learning model, they were not.

Koohestani and colleagues in 2019 used a qualitative method to design an m-Learning model for medical students according to local conditions and contexts ( 38 ). This model was one of the first models of m-Learning in medical education in Iran. In this study, two of the main stakeholders of education, students and teachers, were considered. Also, the individual characteristics of students for accepting m-Learning were mentioned in detail. One of the features of this model is that, in addition to the student's interaction with peers and teachers, interactions with family are also mentioned. However, this model has not been tested. Another point is that other stakeholders such as the director of the institute, technical experts, etc. are not considered.

According to this model based on the informal experiences of students and teachers of m- learning, the operational aspects of m-Learning implementation, including the design of educational content, content features, and students' assessment and the use of m-Learning along with traditional education have not been considered. The results of this study have been published, and so far it has not been referenced or criticized.

In the fifth model, Aliaño et al. showed the factors influencing the acceptance and intention of using mobile devices as learning resources in medical education ( 39 ). The superiority of this model over other models of technology acceptance is due to its completeness because it is a combination of other models ( 49 , 50 ). This model is presented in a complete and integrated way. It can also predict up to 70% of stakeholder acceptance behavior in the face of innovations and technologies.

In this model, the individual characteristics and demographics of the student are mentioned in detail; students are considered the only stakeholders. Other elements such as learning context, interaction with other stakeholders, use of m-Learning along with traditional education, educational design, mobile content features, as well as student assessment are not mentioned in the design and implementation of m-Learning. In terms of technology, network connection, technical support, and the cost of equipment and facilities are not mentioned.

Lall et al. introduced a modified FRAME model by examining the factors that facilitate or hinder the implementation of m-Learning strategies in medical and nursing education ( 40 ). Khosravi and colleagues also pointed out that all elements of this model are effective in educating paramedical students ( 51 ). This model has an additional loop entitled "Implementation in a Clinical Field." This loop shows that even when mobile devices are introduced for training, factors such as adequate course content, adequate Wi-Fi coverage, and staff training capacity to use m-Learning must be considered. Another feature of this model is that it pays attention to a wide range of student interactions, including interactions with the teacher, peers, patients, other health and content specialists. The study by Abou Shosha is in line with this study ( 52 ). However, this model does not mention the features of mobile content as well as student assessment. Despite special attention to the technology aspect, it does not mention issues such as ethical concerns about patient privacy and data security. There is a concern that this type of education may jeopardize the well-being of patients.

In the seventh model, Kucuk and colleagues ( 41 ) proposed a model of medical students' behavioral intent to use planned learning based on the theory of planned behavior (TPB). Azizi et al. and Ju et al., also acknowledged our model ( 53 , 54 ). Since the most important reference groups in education are faculty members and students, the readiness of educators and students and their views on m-Learning are crucial for successful implementation of the learning system mentioned in this model.

However, factors such as the performance of various m-Learning activities independent of the medical course by students may limit the use of this model to design the environment and implement effective m-Learning in medical education. Another point is that in this model, factors such as educational content design, content features, and student assessment, and the use of m- learning along with traditional education, the context of learning are not considered. Also, the technology aspect is generally neglected in this model.

Mosalanejad and colleagues presented a model based on the factors affecting m-Learning ( 42 ). One of the strengths of this model is that in designing the model Baghcheghi et al. confirmed this result ( 55 ), in addition to using the elements of two important models in the acceptance of m-Learning (TAM, FRAME).

Another feature of this model is that the needs of students, as one of the main stakeholders, are included. No mentioning of other stakeholders, use of m-Learning along with traditional education, factors such as educational design, mobile content, student assessment, technical support, ethical concerns about patient privacy, and data security are also overlooked in this model.

### K-ASK3 model: Models of authors

The eight models discussed above point to some of the features of m-Learning, such as usability, collaboration, and flexibility, while ignoring other important features. The key features of each model were extracted and integrated into the new model for a successful design and implementation of m-Learning. This new model is called K-ASK3. The K-ASK3 model contains all the elements of the other models and has the necessary comprehensiveness. This model includes three main elements of m-Learning: 1. Stakeholders, 2. Interaction, and 3. Technology, influenced by external factors including mobiquette, legitimacy, and awareness of m-Learning. The three main elements refer to the principles of m-Learning pedagogy. 

The first element is stakeholders. Students, teachers, peers, education administrators, educational designers, and technical experts, family or caregivers, patients, and other health professionals are stakeholders in m-Learning and have a significant impact on its successful design and implementation. These people communicate and collaborate using the flexibility offered by mobile technologies ( 33 , 37 - 42 , 56 - 57 ).

The second element is interaction. It includes the educational aspect of m-Learning and interactions between people, devices, and systems. Also, it refers to the characteristics of m-Learning that help the students and teachers to interact with each other in terms of cooperation, blended learning, educational design of m-Learning (content, assessment) in the field ( 21 , 33 , 38 , 40 , 42 , 58 ).

The third element is technology. Technology provides access to learning resources anywhere and anytime. In learning environments, technology plays a mediating role in improving learning comprehension. This element shows the features associated with mobile devices, including network connectivity, flexibility, usability, technical support, reliability, and costs associated with the technology ( 33 , 37 , 38 , 40 , 42 , 57 ).

Other important components of the model introduced above are external factors.

1-Mobiquette: To maintain the confidentiality of patient information, teaching behavioral etiquette of using mobile devices in the clinic to medical students is a necessity ( 15 , 21 , 33 ).

2-Legitimacy: The medical education institute supports the use of mobile resources in different places ( 21 ).

**Figure 2:**
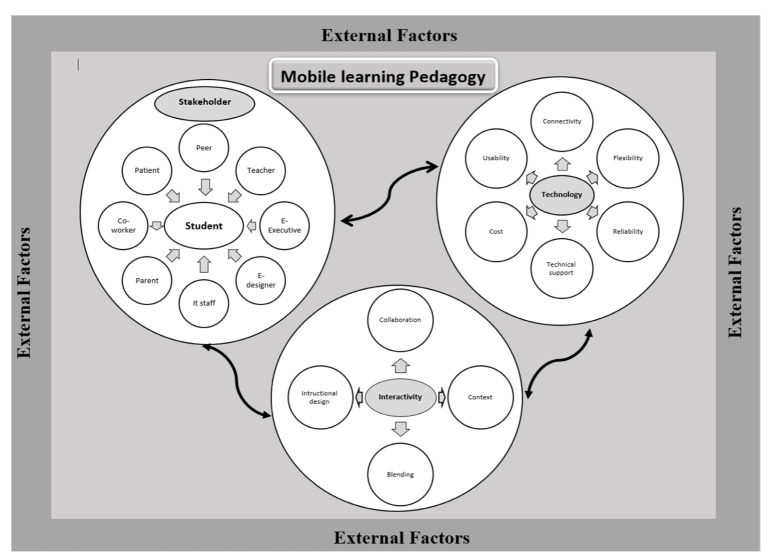
K-ASK3 model of m-Learning in medical education

3-Awareness of m-Learning: Awareness of students and faculties of m-Learning and its benefits affect their intention to use this type of education ( 33 , 41 )(Figure 2).

### Strengths and limitations

 One of the limitations of this study is the limited number of studies in medical sciences and the lack of a similar structure or model to guide the study. Due to the use of only English and Persian articles, some valid documents may not be included in this study. The main focus of the study was on innovative conceptual interpretation of researchers. Another limitation is the low sensitivity of the searches. For this limitation, the authors used experienced researchers. On the other hand, our study has some strengths; we mentioned the Iranian model of m-Learning in our study and used the researchers’ expertise and experience in assessing the quality to increase scientific rigor.

### Future research

Future research can explore the impact of m-Learning on the acquisition of knowledge, attitudes, and skills of medical students in the COVID-19 and post-COVID eras, and its role in theoretical and clinical education.

## Conclusion

The results of this study will be an important contribution to the knowledge collection in the field of m-Learning in medical education. Reviewing the models shows that each model tries to explain a part of the m-Learning strategies that have not been represented in other models. Therefore, we introduce a comprehensive model of m-Learning (K-ASK3 model) that includes specific characteristics of strategies in the medical education context. The K-ASK3 model contains all the elements of the other models. This model includes three main elements of m-Learning: 1. Stakeholders, 2. Interaction, and 3. Technology, influenced by external factors including mobiquette, legitimacy, and awareness of m-Learning. Paying attention to the elements of this model to change the educational policies of institutions may play an important role. Faculty development for using m-Learning should be included in the work program of educational institutions.

## Acknowledgement

The authors would like to thank their colleagues, who provided them with supplemental data.

## Authors' contribution

M.K, M.M.S, S.A, P.K, M.K, N.Kh contributed to the conception and design of the work; the acquisition, analysis, or interpretation of data for the work. All Authors contributed in drafting and revising the manuscript critically for important intellectual content. All authors have read and approved the final manuscript and agree to be accountable for all aspects of the work in ensuring that questions related to the accuracy or integrity of any part of the work are appropriately investigated and resolved.

## Conflict of Interest:

None declared.
